# Antimicrobial Potential of Different Isolates of *Chaetomium globosum* Combined with Liquid Chromatography Tandem Mass Spectrometry Chemical Profiling

**DOI:** 10.3390/biom13121683

**Published:** 2023-11-21

**Authors:** Marwa S. Goda, Noura El-Kattan, Mohamed A. Abdel-Azeem, Kamilia A. M. Allam, Jihan M. Badr, Nourelhuda Ahmed Nassar, Ahmad J. Almalki, Majed Alharbi, Sameh S. Elhady, Enas E. Eltamany

**Affiliations:** 1 Department of Pharmacognosy, Faculty of Pharmacy, Suez Canal University, Ismailia 41522, Egypt; marwa_saeed@pharm.suez.edu.eg (M.S.G.); gehan_ibrahim@pharm.suez.edu.eg (J.M.B.); 2 Department of Microbiology, Research Institute of Medical Entomology, General Organization for Teaching Hospitals and Institutes, Giza 11562, Egypt; nouraelkattan@yahoo.com; 3 Department of Pharmacognosy, Faculty of Pharmacy and Pharmaceutical Industries, Sinai University, Al-Arish, North Sinai 45511, Egypt; mohamed.abdelazeem@su.edu.eg; 4 Department of Epidemiology, Research Institute of Medical Entomology, General Organization for Teaching Hospitals and Institutes, Giza 11562, Egypt; drkamiliaallam@gmail.com; 5 Department of Clinical Pathology, Al-Sahel Teaching Hospital, Cairo 11697, Egypt; nourelhudanassar@yahoo.com; 6 Department of Pharmaceutical Chemistry, Faculty of Pharmacy, King Abdulaziz University, Jeddah 21589, Saudi Arabia; ajalmalki@kau.edu.sa (A.J.A.); maaalharbi1@kau.edu.sa (M.A.); 7 Department of Natural Products, Faculty of Pharmacy, King Abdulaziz University, Jeddah 21589, Saudi Arabia

**Keywords:** antimicrobial, MDR, MIC, *Chaetomium*, LC-MS/MS, public health, drug discovery

## Abstract

The antimicrobial resistance of pathogenic microorganisms against commercial drugs has become a major problem worldwide. This study is the first of its kind to be carried out in Egypt to produce antimicrobial pharmaceuticals from isolated native taxa of the fungal *Chaetomium*, followed by a chemical investigation of the existing bioactive metabolites. Here, of the 155 clinical specimens in total, 100 pathogenic microbial isolates were found to be multi-drug resistant (MDR) bacteria. The *Chaetomium* isolates were recovered from different soil samples, and wild host plants collected from Egypt showed strong inhibitory activity against MDR isolates. *Chaetomium* isolates displayed broad-spectrum antimicrobial activity against *C. albicans,* Gram-positive, and Gram-negative bacteria, with inhibition zones of 11.3 to 25.6 mm, 10.4 to 26.0 mm, and 10.5 to 26.5 mm, respectively. As a consecutive result, the minimum inhibitory concentration (MIC) values of *Chaetomium* isolates ranged from 3.9 to 62.5 µg/mL. Liquid chromatography combined with tandem mass spectrometry (LC-MS/MS) analysis was performed for selected *Chaetomium* isolates with the most promising antimicrobial potential against MDR bacteria. The LC-MS/MS analysis of *Chaetomium* species isolated from cultivated soil at Assuit Governate, Upper Egypt (**3**), and the host plant *Zygophyllum album* grown in Wadi El-Arbaein, Saint Katherine, South Sinai (**5**), revealed the presence of alkaloids as the predominant bioactive metabolites. Most detected bioactive metabolites previously displayed antimicrobial activity, confirming the antibacterial potential of selected isolates. Therefore, the *Chaetomium* isolates recovered from harsh habitats in Egypt are rich sources of antimicrobial metabolites, which will be a possible solution to the multi-drug resistant bacteria tragedy.

## 1. Introduction

Due to the explosive increase in microorganisms resistant to a number of antibacterial and antifungal compounds over the past decade, managing infections in both community and hospital settings has become more difficult and has grown to be a substantial worldwide worry [1,2]. Clinical routine frequently links infections to resistant microorganisms in hospital settings; however, they can also happen in the community as a result of selective pressure from antibiotic use. Antimicrobial resistance (AMR) is on the rise in part due to the environment’s overuse of antimicrobials [3]. Furthermore, the growth, spread, and persistence of multi-drug-resistant (MDR) bacteria pose an increasing hazard to the health of people, animals, and the environment [4]. MDR bacteria often exhibit resistance to three or more antibiotics. In the end, the discovery of antibiotics has lagged due to the advent and evolution of MDR [4]. Natural products remain a promising source of new efficacious antimicrobials to counter increasing resistance and prevent the emergence of multi-drug resistant bacteria [5]. Novel antimicrobial metabolites from endophytic fungi are now becoming an alternative option to overcome the increasing levels of drug resistance by human pathogens. An outstanding model that is utilized as a biotechnological tool in many disciplines related to bioactive molecules is the genus *Chaetomium* [6].

Genus *Chaetomium* is primarily found in soil and organic compost, but some species of *Chaetomium* have also recently been isolated from coral, soft coral, and marine algae [7]. *Chaetomium* species are worldwide species that often live on plant debris and are found in soil and the air. Various metabolites were characterized from different taxa of *Chaetomium* and showed cytotoxicity against a panel of human solid tumour cell lines: NCI-H460 lung cancer, MCF-7 breast cancer, SF-268 brain cancer, and different prostate cancer cell lines (PC-3, LNCaP, and DU-145) as mentioned by [8]. Some members of *Chaetomium* isolates have been identified to produce a number of antimicrobial compounds with antagonistic mechanisms against other pathogenic fungi [9].

From a chemical perspective, more than 350 species of the genus *Chaetomium* have been extensively investigated to find new bioactive secondary metabolites with unique structures from mycelium or spores [10,11]. More than 200 secondary metabolites were isolated and identified from *Chaetomium globosum*, with cytotoxicity, antimicrobial, antimalarial, anticancer, and antiviral actions [12]. Among them, emodins, chrysophanols, chaetoglobosins A–G, isochaetoglobosin, chetomin, azaphilones, chaetoviridins, terpenoids, chaetoglobosins, tetramic acids, steroids, xanthones, diketopiperazines, bis (3-indolyl)-benzoquinones, azaphilones, anthraquinones, pyranones, and orsellides were identified [12,13]. Various studies carried out by various researchers since 1944 on the antibacterial potential of *Chaetomium* have shown that the Gram-positive and Gram-negative bacteria *Escherichia coli* and *Staphylococcus aureus* were particularly susceptible to the antibacterial effects of *Chaetomium* metabolites [14] Additionally, cochliodones were recovered from *C. globosum* species, showing antifungal activities in low doses (1–10 g/mL) against various genera of microfungi, including *Botrytis allii* and *Fusarium moniliforme.* The polysaccharides produced by *Chaetomium globosum* CGMCC 6882 recorded anticancer properties against human lung cancer A549 cells [15].

Until now, neither antimicrobial activity nor chemical profiling of *Chaetomium* species isolated from different ecological habitats in Egypt have been studied. Therefore, this study aimed to assess the antimicrobial potentialities of secondary metabolites of native *C. globosum* against MDR microbes, followed by the rapid identification of natural products using liquid chromatography coupled with tandem mass spectrometry (LC-MS/MS) of the most promising taxa.

## 2. Materials and Methods

### 2.1. Fungal Isolation and Identification

#### 2.1.1. Collection of Samples

Different samples from cultivated and desert soils were collected from various habitats and locations as described in Table 1.

Samples of the most common medicinal plant species in the Saint Katherine protectorate (SKP), Sinai-Egypt, were collected. The taxa of medicinal plants were *Artemisia herba-alba* Asso; *Achillea fragrantissima* (Forssk) Sch.; *Ballota undulata* (Sieber ex Fresen.) Benth.; *Chiliadenus montanus* (Vahl) Brullo; *Alkanna orientallis* (L.) Boiss.; *Origanum syriacum* L. *Peganum harmala* L.; *Phlomis aurea* Decne; *Teucrium polium* L.; *Verbascum sinaiticum* Benth.; *Thymus decussates* Benth.; *Tanacetum sinaicum* (Fresen.) Delile ex K. Bremer & Humphries; *Zygophyllum album*; and *Adiantum capillus-veneris* L. (Table 2). All plant materials were collected for scientific purposes under permission from SKP (#1312-SKP), and no threatened or endangered species were included in this study.

Also, mangal samples of *Avicennia marina* (Forssk.) Vierh., *Ziziphus spinosus* Semen, and *Adiantum capillus-veneris* L. were collected from Safaga, Nuwieba, and Port Fouad, respectively (Table 2).

All collected samples were transported in sterilized polyethylene bags to the lab, where they were later plated out.

#### 2.1.2. Isolation and Preservation of Mycobiota

For the isolation of terricolous taxa of *Chaetomium*, an alcohol immersion technique was applied according to [16]. Regarding the isolation of endobiotic (endophytic) fungi, the aerial parts (leaf and stem) of each plant were washed in running water, cut into small pieces, and surface-sterilized by dipping in 75% ethanol (EtOH) (*v*/*v*) for 1–5 min depending on the plant thickness, and then dipped in 0.05 g/mL sodium hypochlorite (NaOCl) solution (*v*/*v*) for 3–5 min, followed by two rinses in sterile distilled water [17].

Different types of isolation media, including malt extract agar (MEA), Czapek’s yeast extract agar (CYA), and potato dextrose agar (PDA) supplemented with rose Bengal (1/15,000) as bacteriostatic and chloramphenicol (50 ppm) as bactericide [18], were used for the isolation of fungi. Plates were plated and then incubated for 10 days at 27 °C.

#### 2.1.3. Phenotypic Identification of *Chaetomium* Isolates

The following identification keys were utilized to identify the isolated culturable *Chaetomium* isolates phenotypically down to the species level on standard medium: for Ascomycetes [19] and for *Chaetomium* [20]. Carl Zeiss amplival microscope (GmbH, Germany) was used to examine the microscopic characteristics. All name corrections, authorities, and taxonomic assignments of recorded species in the present study were checked against the Index Fungorum database (www.indexfungorum.org) (accessed on 4 May 2023), followed by the 10th edition of Ainsworth and Bisby’s Dictionary of the Fungi [21]. Heatmaps were generated using the ggplot2 package in R software Ver. 2023.09.0+463 for MacOS. 

### 2.2. Culture of Chaetomium Isolates and Extraction of Their Active Metabolites

#### 2.2.1. Subcultures of Pure Isolates of *C. globosum*

A pure culture of *Chaetomium* isolates was grown on an oatmeal agar medium for 14 days at 27 °C. Forty grams of rice were mixed with 100 mL of distilled water in a 500 mL Erlenmeyer flask (three replicas for each isolate) before being autoclaved [22]. Under sterile conditions, five discs from each isolate were inoculated into each flask by using a cork borer (1 cm in diameter). All flasks were fermented and incubated for 21 days at 27 °C.

#### 2.2.2. Extraction of Active Metabolites

The fermented flasks for each isolate (3 flasks) were homogenized in 300 mL of deionized water (100 mL per flask) using a high-speed blender (Tornado electric blender, 500 Watt, Cairo, Egypt). The resulting mixture was macerated overnight with ethyl acetate in a ratio of 3:1 with 3 repetitions to extract fungal metabolites. The organic layers were then combined and evaporated under reduced pressure at 40 °C to obtain a crude extract.

### 2.3. Assessment of Antimicrobial Activity of Different Isolates of Chaetomium Isolates

#### 2.3.1. Collection of Clinical Samples and Isolation of Microbes

About 155 clinical specimens of blood, sputum, wound, pus, urine, and cerebrospinal fluid (CSF) were taken from patients suffering from different infections, admitted to El-Sahel Teaching Hospital, the general organization for teaching hospitals and institutes (GOTHI), Cairo, Egypt. Various specimens were taken from patients whose infections were determined to be present based on clinical manifestations. The patients were of both sexes, with ages ranging from 19 to 80 years old. For each patient, clinical and laboratory data were gathered and recorded. All laboratory procedures were carried out in accordance with the Clinical and Laboratory Standards Institute’s (CLSI) guidelines and regulations (Ethics Code: IME00071). The collected specimens were inoculated onto appropriate isolation culture media, McConkey agar, blood agar and nutrient agar (Oxoid Ltd., Co. ^®^, Nepean, ON, Canada), and then they were incubated at 37 °C. Microbial identification was largely based on colony features and Gram-stain reactions, while final microbial isolate identification was performed using the traditional VITEK 2 compact 15 system (BioMérieux ^®^, Inc., Hazelwood, MO, USA).

#### 2.3.2. Antibiotics Susceptibility Test for Determination of MDR Isolates

All bacterial isolates were tested for antibiotic susceptibility using the standard Kirby–Bauer disk diffusion technique, as defined by Clinical Laboratory Standard Institute guidelines (2018). The most widely used classes of antibiotics, such as tetracyclines, aminoglycosides, penicillins, cephalosporins, fluoroquinolones, and carbapenems, were employed. These Gram-positive and Gram-negative antibiotics were prepared at the following concentrations: amikacin (30 µg), amoxycillin/clavulanic acid (30 µg), ampicillin/sulbactam (30 µg), cefepime (30 µg), cefotaxime (10 µg), ceftazidime (30 µg), gentamicin (10 µg), imipenem (10 µg), meropenem (10 µg), tigecycline (15 µg), ciprofloxacin (5 µg), tetracycline (30 µg), levofloxacin (5 µg), cefoxitin (30 µg), and doxycycline (5 µg). All antibiotics were purchased from Oxoid ^®^, Basingstoke, UK. Multi-drug-resistant (MDR) microbes have been described as those that are resistant to at least one antibiotic in at least three antimicrobial classes [23].

#### 2.3.3. Screening of Antimicrobial Activity of *Chaetomium* Taxa against MDR Isolates

The stock solutions of 10 crude extracts of *Chaetomium* taxa were reconstituted using 10% dimethyl sulfoxide (DMSO), and the extracts were diluted to a concentration of 50 mg/mL. They were screened for their antibacterial and antifungal activities against 100 pathogenic microbial isolates, including Gram-positive bacterial isolates (*Staphylococcus aureus*, *Enterococcus faecalis*, and *Streptococcus pyogenes*), Gram-negative bacteria isolates (*Acinetobacter baumannii*, *Escherichia coli*, *Klebsiella pneumoniea*, *Proteus mirabilis*, *Pseudomonas aeruginosa*, and *Serratia marcescens*), and *Candida albicans*. About 20 μL/disc solvent extract with concentrations of 50 mg/mL was soaked in each sterile Whatman disc with a diameter of 6 mm and allowed to dry before being placed on the inoculated media. Bacterial strains (10^8^ CFU) were seeded into Mueller–Hinton sterile agar plates, while *Candida albicans* were seeded into Sabouraud dextrose agar media. As a negative control, dimethyl sulfoxide (DMSO) was used. The zones of growth inhibition surrounding the disks were measured using a calibrated ruler after 48 h for fungi at 28 °C and after 18 to 24 h of incubation at 37 °C for bacteria. The sensitivities of the microorganisms to the fungal extracts were assessed by measuring the sizes of inhibitory zones in millimetres on the agar surface surrounding the disks, and those with no zones were labelled negative. Results are presented as the mean value (±standard deviation). Minimum inhibitory concentrations (MIC) were determined for fungal crude extracts that showed inhibition activity against the test microorganisms.

#### 2.3.4. Determination of Minimum Inhibitory Concentrations (MIC) of Active Isolates

The MIC was obtained with the microdilution technique using a 96-well microtitre plate according to the Clinical Laboratory Standards Institute (CLSI, 2015). A volume of 100 µL of two-fold diluted extracts in Muller–Hinton broth was introduced in the wells of the plate. Thereafter, 100 µL of inoculum standardized at 0.5 Macfarland and extracts were added to each well to a final volume of 200 μL containing the test substances except for the blank column for sterility control. The concentrations of the test substances ranged from 0.5 to 500 µg/mL. For bacterial and fungal pathogens, the plates were incubated at 37 °C for 24 h and 28 °C for 48 h, respectively. Turbidity was considered an indicator of growth, and the MIC was determined as the lowest concentration that inhibits observable bacterial growth.

### 2.4. Metabolic Profiling of Selected Chaetomium Isolates Using LC-MS/MS

The metabolic profiling and composition were established using (HPLC/triple-TOFMS and MS) [24,25]. The ethyl acetate extracts of selected isolates of *C. globosum* (3 and 5) were dissolved in water, methanol, and acetonitrile (50:25:25) mixture to afford a solution with a concentration of 0.5 mg/mL. The prepared solution was centrifuged, and then 50 µL was picked up and completed to 1000 µL with water, methanol, and acetonitrile (50:25:25). Ten µL was injected in both positive and negative modes. The LC/Triple-TOF-MS/MS analysis was conducted using an ExionLC system (AB Sciex, Framingham, MA, USA) with an autosampler system, an in-line filter disk precolumn (0.5 µm × 3.0 mm, Phenomenex, Torrance, CA, USA), and an X-select HSS T3 column (2.5 µm, 2.1 × 150 mm, Waters Corporation, Milford, MA, USA) sustained at 40 °C. The mobile phase consisted of 5 mM ammonium format buffer in 1% methanol, with the pH adjusted to 3.0 and 8.0 for positive and negative modes, respectively. The mobile phase was gradually eluted by raising the acetonitrile concentration over 20 min, and then a steady period of 4 min, followed by a reduction in the acetonitrile concentration over 3 min at a constant flow rate of 0.3 mL/min. This compartment was linked to a Triple TOF^TM^ 5600+ system (AB SCIEX, Concord, ON, Canada) to monitor the analytes’ MS/MS transitions. The detected metabolites were recognized by their *m*/*z* and MS/MS transitions to those reported in previously documented databases. The mass accuracy was calculated as follows: [measured mass-expected mass/expected mass] × 10^6^ and expressed in parts per million (ppm) error [26,27,28]. Mzmine ID, retention time, adduct formula, and molecular formula were also detected.

## 3. Results and Discussion

### 3.1. Fungal Sources and Identification of Chaetomium sp.

**Figure 1:**
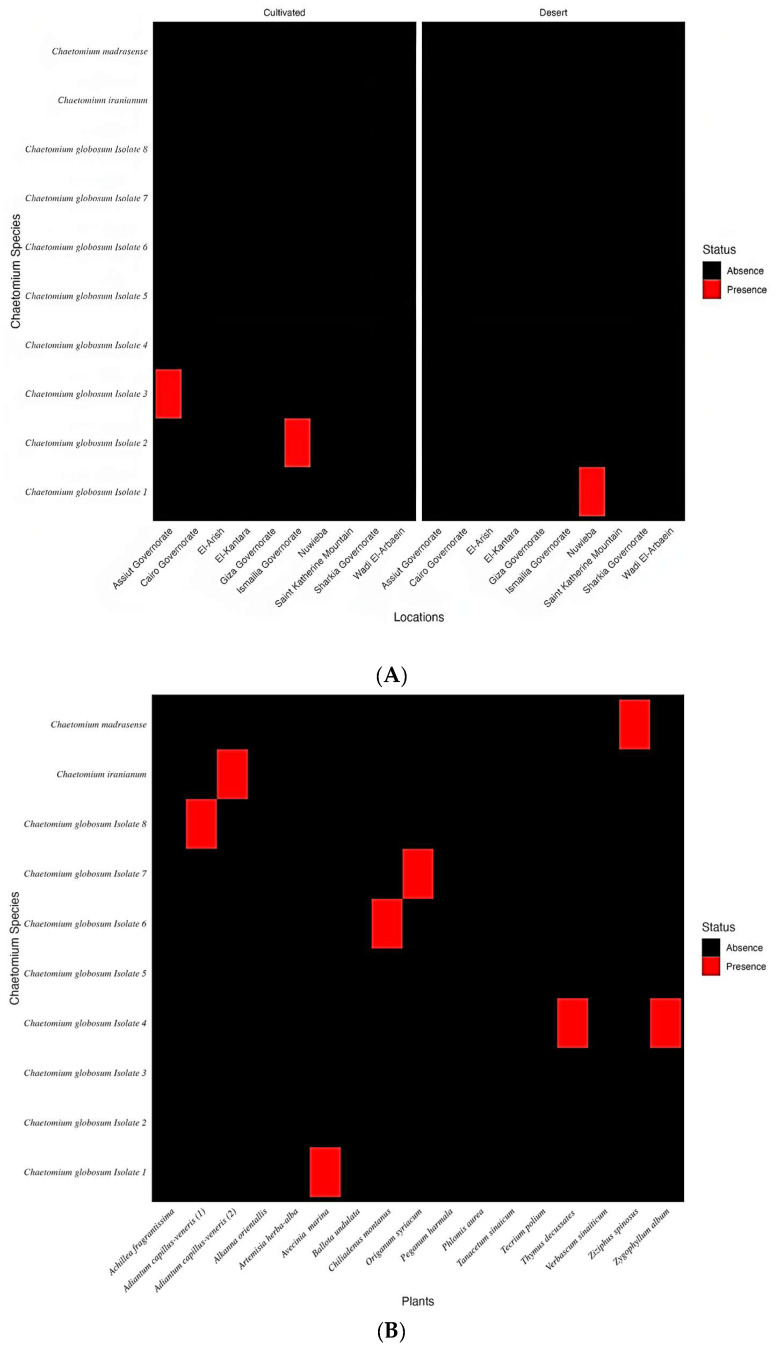
Heatmap of presence/absence of *Chaetomium* isolates per source, soil (**A**); plants (**B**).

As shown in Figure 1, it was possible to encounter as many as 10 isolates of *Chaetomium* recovered during the entire study from all habitats. All identified taxa were deposited in the Suez Canal University Fungarium at the Botany and Microbiology Department, Faculty of Science (https://ccinfo.wdcm.org/collection/by_id/1180 (accessed on 15 November 2022). Out of the 27 collected samples that were plated out, a total of 10 teleomorphic isolates were morphologically identified as members of Chaetomiaceae (Table 3).

**Figure 2:**
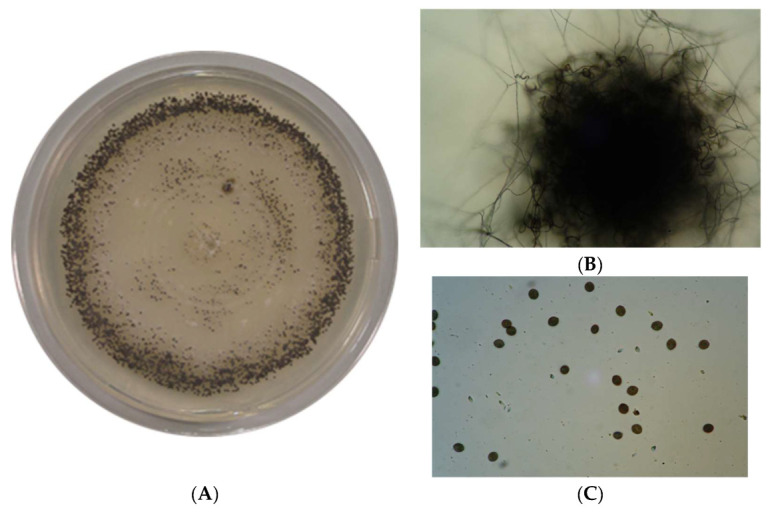
*Chaetomium globosum*: (**A**) colony; (**B**) ascomata showing peridial hairs; (**C**) Limoniform ascospores.

*Chaetomium globosum* colonies are characterized by a pale or olivaceous aerial mycelium with yellow, greyish green, green, or red exudates. Ascomata mature within 7–9 days (Figure 2A), showing olivaceous, grey-green, or brown colour in reflected light. The colonies are superficial, spherical, ovate, or obovate in shape with a brown intricate ascomatal wall and 2–3.5 μm broad, having ostiolate pores with a 175–280 μm width. The ascomatal hairs are numerous, usually unbranched, flexuous, undulate or coiled, often tapering, septate, brownish, 3–4.5 μm broad at the base, and up to 500 μm long (Figure 2B). Asci are clavate or slightly fusiform, stalked, 30–40 × 11–16 μm, 8-spored, and evanescent. Ascospores are limoniform, usually biapiculate, bilaterally flattened, brownish when mature, thick-walled, and containing numerous droplets (9–12 × 8–10 × 6–8 μm) with an apical germ pore (Figure 2C).

### 3.2. Antimicrobial Assessment of Chaetomium Isolates

#### 3.2.1. Microbial Isolates and Their Antibiotic Resistance Pattern

Of the 155 clinical specimens in total, 100 pathogenic microbial isolates were obtained and found to be MDR (Table 4). According to [29], Gram-negative isolates were more commonly found than Gram-positive ones: *Staphylococcus aureus*, *Candida albicans*, *Enterococcus faecalis*, and *Streptococcus pyogenes*.

The antibiotics that are used for repeated empirical treatment might be the reason for the development of high antibiotic resistance. The antibiotic resistance patterns of all isolated and identified strains are shown in Table 5.

*S. aureus* strains were found to be 100% resistant to amoxycillin/clavulanic acid, ampicillin/sulbactam, cefepime, and cefotaxime; however, about 100% of *S. aureus* strains were sensitive to cefoxitin. *S. aureus* is one of the most common multi-drug-resistant bacterial pathogens, causing different infections [30].

Also, *E. faecalis* strains showed 100% resistance to amikacin, ampicillin/sulbactam, cefepime, cefotaxime, ceftazidime, and cefoxitin, while it showed 100% sensitivity to tigecycline, similar to results found by [31]. The multi-drug resistance of *E. faecalis* was observed due to its ability to acquire and transfer antibiotic resistance genes [32].

Moreover, 100% of *S. pyogenes* strains were resistant to amoxycillin/clavulanic acid, ampicillin/sulbactam, cefepime, cefotaxime, ceftazidime, and cefoxitin. On the other hand, tigecycline, imipenem, and meropenem were completely active against 100% of *S. pyogenes* strains; such results were represented by [33]. In earlier studies, [34] reported that carbapenems are still sensitive to upcoming resistance from both Gram-positive and Gram-negative bacteria.

Similar to the results of the study stated by [35], all *A. baumannii* strains showed resistance to cefoxitin and doxycycline; 85.7% of the same strain showed resistance to ampicillin/sulbactam, cefepime, ceftazidime, and ciprofloxacin.

*E. coli* strains were mostly resistant to cefoxitin, amoxycillin/clavulanic acid, ampicillin/sulbactam, ceftazidime, gentamicin, cefotaxime, and ciprofloxacin with a percentage of 100%, 90.9%, 90.9%, 90.9%, 90.9%, 81.8%, and 81.8%, respectively. This was similar to the previous study by [36,37].

*K. pneumoniae* is one of the WHO’s list of antibiotic-resistant pathogens that necessitate the development of new antibiotics to combat them [38]. It is a well-known nosocomial pathogen that has recently emerged as an MDR and pan-drug-resistant issue [39]. In our study, *K. pneumoniea* found high antibiotic resistance to amoxycillin/clavulanic acid (100%), ampicillin/sulbactam (100%), levofloxacin (100%), cefotaxime (90%), cefoxitin (90%), ciprofloxacin (80%), tetracycline (80%), amikacin (73.3%), ceftazidime (73.3%), gentamicin (70%), cefepime (70%), doxycycline (60%), and tigecycline (60%), while 70% and 66.7% were susceptible to imipenem and meropenem, respectively, similar results reported by [40].

*P. mirabilis* shows varying degrees of resistance to antibacterial drugs, with the highest resistance (100%) to cefotaxime and cefoxitin, followed by (80%) resistance to ampicillin/sulbactam, ciprofloxacin, gentamicin, and levofloxacin, while 60% resistance was recorded to amoxycillin/clavulanic acid, cefepime, ceftazidime, doxycycline, and tetracycline; furthermore, 80% of this strain were sensitive to imipenem, meropenem, and tigecycline; these findings are in agreement with a previous study by [41]. *P. mirabilis* may cause a number of opportunistic and nosocomial infections due to their virulence factors [42].

*P. aeruginosa* isolates showed susceptibility to meropenem, imipenem, and tigecycline, 66.7%, 88.9%, and 88.9%, respectively. In contrast, the same strain showed 100% resistance to amoxycillin/clavulanic acid, cefepime, cefotaxime and tetracycline, followed by 88.9% resistance to ampicillin/sulbactam, cefoxitin, ciprofloxacin, gentamicin, and levofloxacin. These results are in agreement with those found by [35]. According to [43], *P. aeruginosa* is basically resistant to many antibiotics and is capable of easily acquiring antibiotic resistance. Furthermore, *P. aeruginosa* has a high potential to evolve multi-drug resistance phenotypes [44].

Imipenem, meropenem, and tigecycline were completely effective against *S. marcescens*, while the same strain showed complete resistance to amoxycillin/clavulanic acid, ampicillin/sulbactam, ceftazidime, gentamicin, and levofloxacin. Although *S. marcescens* was previously considered non-pathogenic, this species has emerged as a prominent opportunistic pathogen found in nosocomial infections [45,46]. *S. marcescens,* associated with hospital outbreaks or epidemic events, is commonly resistant to several antibiotics.

Here, all the isolated microbes proved their multi-drug resistance against most tested antibiotics.

#### 3.2.2. The Antimicrobial Activity of *Chaetomium* Isolates against MDR Strains

Because of the emergence and spread of antimicrobial resistance by pathogenic microorganisms to commercial drugs, the current study was carried out to determine the antimicrobial potential of terricolous and endophytic fungi isolated from different habitats. As described in Table 6, all isolates of *Chaetomium* had significant inhibitory activity against Gram-negative bacteria. At the same time, *Chaetomium globosum* isolates (**3**,**4**,**5**,**6**) and *Chaetomium madrasense* (**10**) showed a wide spectrum of antimicrobial activity against Gram-positive and Gram-negative pathogenic microorganisms that were isolated from different clinical samples and showed multi-drug resistance ability.

*C. globosum* isolates demonstrated substantial inhibitory activity against Gram positive bacteria (*S. aureus*, *E. faecalis*, and *S. pyogenes*) with different inhibition zones ranging from 10.4 to 21 mm. Additionally, they offered variable sensitivity against Gram negative bacteria (*A. baumannii*, *E. coli*, *P. mirabilis*, *P. aeruginosa*, and *S. marcescens*) with inhibition zones of 11.3 to 26.5 mm.

**Figure 3:**
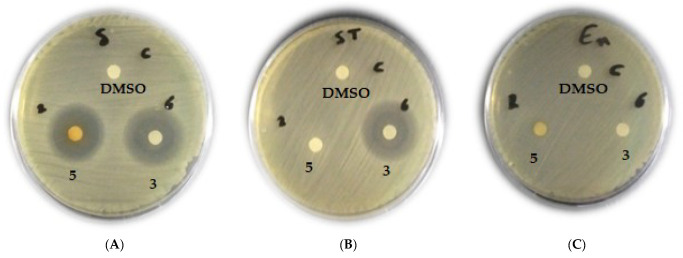
Antimicrobial activity of crude extracts of *C. globosum* isolated from cultivated soil at Assuit (**3**) and *Zygophyllum album* (**5**) against Gram-positive bacteria: *S. aureus* (**A**); *S. pyogenes* (**B**); *E. faecalis* (**C**), using DMSO as a negative control.

**Figure 4:**
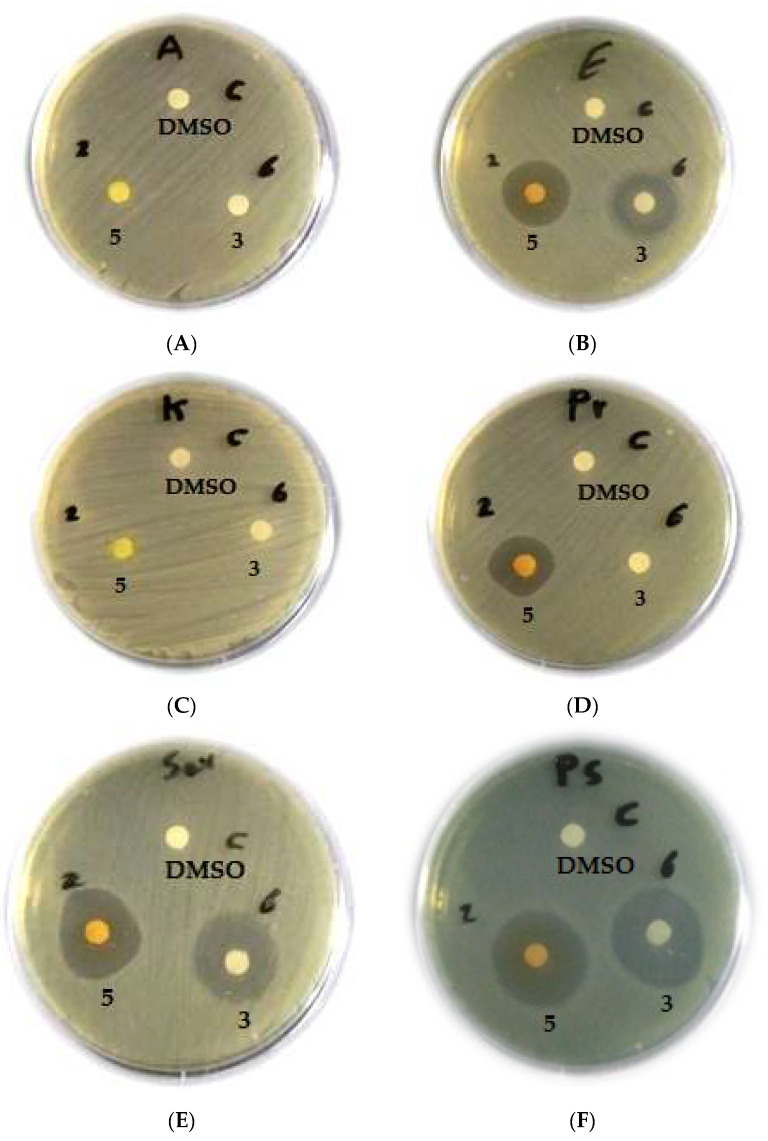
Antimicrobial activity of crude extracts of *C. globosum* isolated from cultivated soil at Assuit (**3**) and *Zygophyllum album* (**5**) against Gram-negative bacteria: *A. baumannii* (**A**); *E. coli* (**B**); *K. pneumonia* (**C**); *P. mirabilis* (**D**); *S. marcescens* (**E**); *P. aeruginosa* (**F**), using DMSO as a negative control.

The maximum ranges of zones of inhibition were provided by *C. globosum* isolated from cultivated soil in Assiut Governorate (**3**) and *Zygophyllum album* from Wadi El-Arbaein (**5**), as represented in Figure 3 and Figure 4.

**Figure 5:**
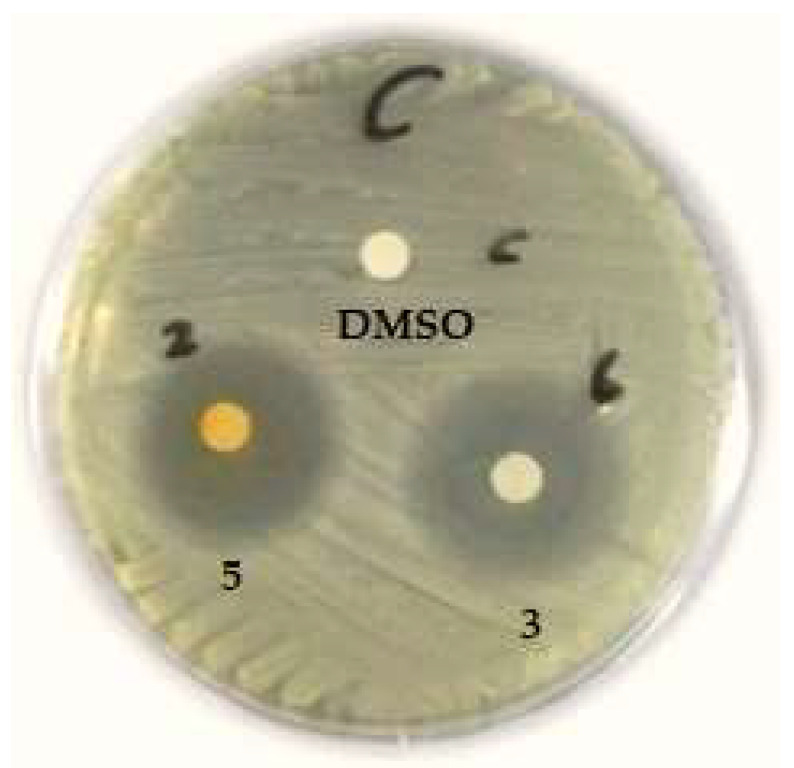
Antimicrobial activity of crude extract of *C. globosum* isolated from cultivated soil at Assuit (**3**) and *Zygophyllum album* (**5**) against *C. albicans*, using DMSO as a negative control.

In comparison to other taxa, both isolates (**3** and **5**) displayed the highest inhibition zones against *C. albicans* (Figure 5).

Obviously, the *C. globosum* isolated from cultivated soil in Assiut Governorate (**3**) and from host plant *Zygophyllum album* (**5**) showed strong antimicrobial activity against most tested pathogenic microorganisms; our findings were similar to previous studies reported by [15,47]. On the other hand, *Chaetomium iranianum* (**7**) recorded no inhibitory activity with *S. aureus*, *E. faecalis* and *S. pyogenes*, *A. baumannii*, *K. pneumonia*, *P. mirabilis*, and *C. albicans*, but it showed a high inhibitory effect against *E. coli*, *P. aeruginosa*, and *S. marcescens* with inhibition zones of 18.4, 25.2, and 26.0 mm, respectively. *C. madrasense* (**10**) was effective against Gram-positive bacteria, and *C. albicans*, but no inhibition zones were observed with *A. baumannii*, *K. pneumonia* and *P. mirabilis* strains.

#### 3.2.3. Minimum Inhibitory Concentration (MIC) of Fungal Extracts

The minimum inhibitory concentration (MIC) of crude extracts of *Chaetomium* was found to range from 3.9 to 62.5 µg/mL, depending on the fungal extracts and the tested microorganisms (Table 7).

Crude extracts of *Chaetomium* showed significant activity against Gram-negative bacteria and *Candida albicans*, followed by Gram-positive bacteria. Our findings are in agreement with [15,48], but they are lower than those found by [49,50].

Many antimicrobial compounds isolated from endophytic fungi can be used for pharmaceutical, medicinal, and agricultural applications [51]. Examples of these secondary metabolites are chaetocin, ergosterol, and terpenes [52,53]. These variations in susceptibility might be linked to the type of isolates, the nature and concentration of antimicrobial compounds present in their extracts, as well as their mechanism of action on various microorganisms. Regarding the promising antimicrobial activity of the two isolates of *Chaetomium* (**3**, **5**), they were subjected to further chemical studies.

### 3.3. LC-MS/MS Metabolic Profiling of Crude Extracts of C. globosum Isolates **3** and **5**

For the rapid identification of the bioactive metabolites correlated to the promising antimicrobial activity of isolates of *C. globosum* (**3** and **5**), LC-MS/MS analysis was conducted. The physical separation of various metabolites depends on their affinity for the reversed stationary phase. At the same time, their identification is based on the calculation of mass error accuracy and detection of the daughter mass fragments [24,25].

#### 3.3.1. Identification of Natural Metabolites in the Crude Extract of *C. globosum* Soil Taxon Recovered from Cultivated Soil in Assiut Governorate, Upper Egypt, (**3**) Using LC-MS/MS Technique

Alkaloids are the major metabolites detected in the *C. globosum* soil taxon recovered from cultivated soil in Assiut Governorate, as shown in Table 8.

**Figure 6:**
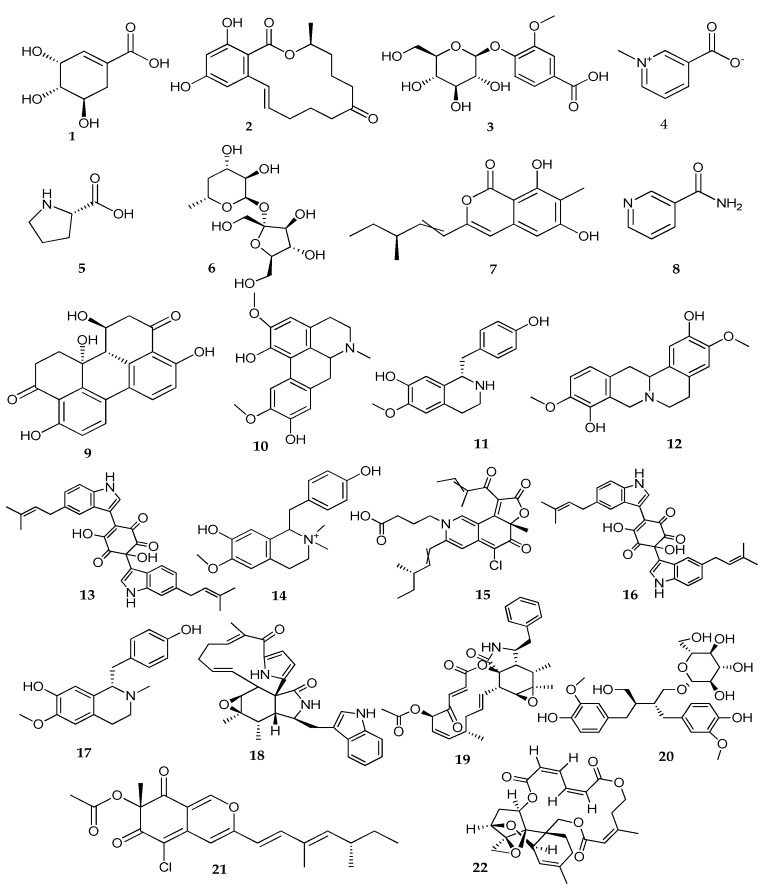
Chemical structures of metabolites detected by LC-MS/MS listed in Table 8.

In terms of alkaloids being the major detected metabolites as shown in Figure 6, trigonelline has been shown to have antibacterial, antiviral, hypoglycemic, hypolipidemic, neuroprotective, memory-improving, and anti-tumour activities, in addition to reducing platelet aggregation [73]. Aporphine isoboldine alkaloid was found to have an antifungal effect against *Tricophyton rubrum* [74]. Coclaurine is an isoquinoline alkaloid that has antiviral properties [75] and cytotoxic effects against human colon cancer (HCT116), human breast cancer (MCF7), and human liver cancer (HEPG-2) cells, with corresponding IC_50_ values of 8.233, 15.345, and 1.674, respectively [76]. Scoulerine is a benzylisoquinoline alkaloid showing inhibitory activity against acetylcholinesterase (anti-AChE), tumour necrosis factor-alpha (anti-TNF-α), and the bacterial strain *Helicobacter pylori* [77]. Numerous fungi from the genera *Phoma*, *Helminthosporium*, *Zygosporium*, *Metarrhizium*, *Chaetomium*, and *Rosellinia* produce cytochalasins [78]. Indole alkaloids, known as chaetoglobinol A and B, were detected; they displayed a hypoglycemic effect via the inhibition of α-glycosidase [66]. Chaetoglobinol A has antibacterial properties against *Bacillus subtilis* [79]. Another azaphilone alkaloid, chaetomugilide A, displayed cytotoxic effects against HepG2 [80] in addition to its antimicrobial property against *Pseudomonas putida* and *Bacillus subtilis* at concentrations of less than 20 μM [81]. Penochalasin A showed cytotoxic activity against the human nasopharyngeal epidermoid tumour KB cell line with an IC_50_ value of 48 μM [82]. Cytochalasin alkaloids can alter cellular shape, prevent cellular activities like cell division, and even trigger apoptosis [83]. Moreover, the antibacterial activity of sclerotiorin against *Micrococcus luteus*, *Bacillus cereus*, Bacillus subtilis, Klebisiella pneumoniae, *E. coli*, Salmonella typhimurium, and *L. monocytogenes* was reported [84].

Other chemical categories were found. Among them is shikimic acid, which is a source of precursors involved in the biosynthesis of aromatic amino acids and phenylpropanoid metabolites. Its antioxidant, anti-inflammatory, antiviral, neuroprotective, and antibacterial properties have been the subject of several investigations. Zearalenone macrolide has a fourteen-membered lactone ring fused to 1,3-dihydroxybenzene, which displays an estrogen-like action via binding to the estrogenic receptors in the ovaries, mammary glands, uterus, or vagina [85]. Vanillic acid is a phenolic acid that has been employed as a food flavouring agent, preservative, anti-inflammatory, antioxidant, and antihypertensive [86,87]. It also showed antimicrobial, anti-filarial, snake venom antagonist properties in addition to its antibacterial action against foodborne pathogens such as *Staphylococcus aureus* and *E. coli* [88,89]. It was previously mentioned that L- proline-rich peptides are prospective medicines to fight multi-drug-resistant microbes [90].

An isocoumarin derivative known as prochaetoviridin A displayed moderate antifungal activity against five pathogenic fungi, including *Sclerotinia sclerotiorum*, *Botrytis cinerea*, *Fusarium graminearum*, *Phytophthora capsici*, and *Fusarium moniliforme*, with inhibition rates ranging from 13.7% to 39.0% [61]. Nicotinamide is an effective antimicrobial agent against the human immunodeficiency virus, *Mycobacterium tuberculosis*, and *Plasmodium falciparum* [91]. Perylenequinone altertoxin I is a pentacyclic aromatic polyketide and exhibited strong cytotoxicity with LC_50_ values (concentration causing 50% inhibition) of 6.43 µM [92]. Altertoxin I was found to be a potential inhibitor of the replication of the HIV-1 virus [93]. Due to the potent antiproliferative, antioxidant, antiestrogenic, and/or antiangiogenic properties of secoisolariciresinol lignan, it has been found to prevent various cancers, including breast, lung, and colon cancers [94]. Verrucarin J is one of the trichothecenes or sesquiterpene metabolites containing a tricyclic skeleton and an epoxide group. Without impacting cell viability, macrocyclic trichothecene verrucarin J suppressed the arenavirus *Junin,* which causes hemorrhagic fever with IC_50_ values in the range of 1.2–4.9 ng/mL [95]. It is worth noting that several cytotoxic agents have been proven to suppress or delay microorganism growth in vitro [96,97].

Now, it is obvious to deduce that the antibacterial property of the crude extract of *C. globosum* soil taxon (**3**) against multi-drug resistant bacteria refers to the antimicrobial activity of the detected metabolites, as indicated above.

#### 3.3.2. Identification of Natural Metabolites in the Crude Extract of *C. globosum* Endophytic Taxon Recovered from *Zygophyllum album* (**5**), Wadi El-Arbaein, Saint Katherine, South Sinai, Using LC-MS/MS Technique

**Figure 7:**
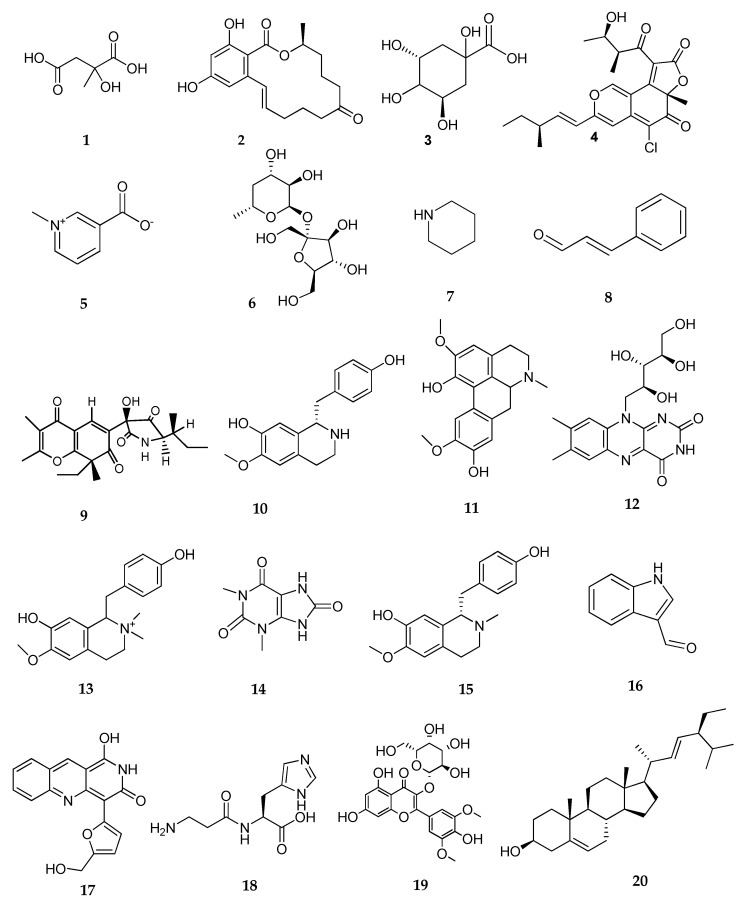
Chemical Structures of metabolites detected by LC-MS/MS listed in Table 9.

In the same manner, it was found that the majority of identified metabolites were chemically classified as alkaloids in the *C. globosum* endophytic taxon recovered from *Zygophyllum album* (**5**) (Table 9, Figure 7).

In terms of detected alkaloids, trigonelline, coclaurine, N-methyl coclaurine, N,N-dimethyl coclaurine, and demethyl isoboldine are common alkaloids in isolates of *C. globosum* (**3** and **5**). Moreover, piperidine has been shown to have antibacterial, antimalarial, anti-inflammatory, analgesic, antioxidant, anti-hypertensive, and antiproliferative actions [109]. 

Citramalic acid is an analog of malic acid, containing an alpha-hydroxyl di-carboxylic group. It is involved in the biosynthesis of the branched-chain amino acid pathway and also in cosmetics production to reduce skin wrinkles [110]. Quinic acid is an alpha-hydroxyl acid that demonstrated strong antibacterial effects on *S. aureus* via lysis of the cell membrane and interfering with cellular metabolism [111]. It also acts as a radioprotective, anti-diabetic, anti-neuroinflammatory, neuroprotective, anti-mutagenic, and anti-inflammatory agent [112].

Chaetoviridins have a pyrone–quinone structure and are typically referred to as azaphilones [113]. Chaetoviridin A has antimalarial, antimycobacterial, antifungal, and cytotoxic activities [114]. The growth of *S. sclerotiorum*, *Rhizoctonia solani*, *Magnaporthe grisea*, and *Pythium ultimum* can be inhibited by chaetoviridin A [61,115].

Several in vitro and in vivo investigations have looked at cinnamaldehyde as a possible substitute for antimicrobial therapy [116]. By inhibiting pathogens such as *Candida* spp., *E. coli*, *Listeria monocytogenes*, *Pseudomonas aeruginosa,* and *Staphylococcus aureus*, cinnamaldehyde has demonstrated a broad spectrum of antimicrobial action [117].

In addition to being essential for sustaining human health, recent research revealed that riboflavin can inhibit or inactivate the growth of a variety of microorganisms, including bacteria, viruses, fungi, and parasites. This suggests that riboflavin may have antimicrobial properties [118,119].

Globosumin is an azaphilone, which is a subclass of fungal polyketide metabolites having a highly oxygenated pyranoquinone bicyclic structure. It showed cytotoxic effects against HepG2 and the lung cancer A549 cell line with an IC_50_ of 6.82 μM, demonstrating its biological activity [103]. At the same time, chaetominedione inhibits p56*^lck^* tyrosine kinase with less damage to healthy cells [120].

The dipeptide carnosine possesses functional characteristics that are unique to muscles and excitable tissues. Recent in vivo and in vitro research has demonstrated that carnosine has anti-inflammatory, metal chelating, antioxidant, and free radical scavenging properties [121].

Syringetin is an *O*-methylated flavonol that has antioxidant and antimicrobial properties. Its broad-spectrum antimicrobial activity was observed against *Staphylococcus aureus*, *Escherichia coli*, *Proteus vulgaris*, *Pseudomonas aeruginosa*, *Candida albicans*, and *Microsporum canis*. It is also able to inhibit the growth of cancer cells via the induction of cell cycle arrest in the G2/M phase and the initiation of apoptosis. Syringetin can inhibit the alpha-glucosidase enzyme, so it can reduce postprandial glycemia [122].

Stigmasterol had strong anti-inflammatory and immunomodulatory effects via a reduction in the production of pro-inflammatory cytokines, nitric oxide, and tumour necrosis factor-α (TNF-α), as well as the suppression of cyclooxygenase-2 (COX-2) [123]. In colitis, stigmasterol has been discovered to up-regulate the intestinal mucosal immune response linked to inflammatory bowel disease by activating the butyrate-PPAR axis [124].

At last, it is important to note that prochaetoviridin A [125], chaetoglobinol A [79], chaetomugilide A [68], chaetoglobinol B [66], globosumin [103], and chaetominedione [120] were previously isolated from *Chaetomium*. According to [81], 19 out of 25 strains of *C. globosum* had chaetoviridin A isolated from their filtrates.

## 4. Conclusions

In conclusion, this study sheds light on the potentiality of native *Chaetomium* isolates, which recorded high antibacterial activity against multi-drug resistant clinical Gram-positive and Gram-negative bacteria. Our findings proved that endophytic and terricolous mycobiota, especially those isolated from extreme environments such as Upper Egypt and Sinai, are considered sustainable sources of antimicrobial bioactive compounds. Their anti-microbial activity may be referred to by several compounds, such as chaetomugilide A, chaetoviridin A, prochaetoviridin A, and chaetoglobinol A, as proven by LC-MS/MS chemical profiling. More research involving the isolation of bioactive compounds, safety profile, nanoformulation, and clinical trials of native fungi in Egypt should be conducted in order to discover new drugs for medical, industrial, and nanotechnology applications.

## Figures and Tables

**Table 1:** Global positioning system (GPS) data of soils’ collection sites.

Soil Type	Site	GPS	
		North	East
1- Desert Soil	Saint Katherine Mountain	28°30′23″	34°02′00″
	Nuwieba	28.9°73′44.4″	34.6°53′43.3″
	Wadi El-Arbaein	28°33′00″	33°58′11″
	El-Arish	31°11′10″	33°50′25″
	El-Kantara	30°51′01″	32°16′33″
2- Cultivated Soil	Ismailia Governorate	30°40′00″	32°20′21″
	El-Sharkia Governorate	30°45′00″	31°50′12″
	Assiut Governorate	27.1°80′96″	31.18°36′8″
	Giza Governorate	30°00′20″	31°10′37″
	Cairo Governorate	30°01′11″	31°20′34″

° Degrees; ′ minutes; ″ seconds.

**Table 2:** GPS data of collection sites of host plants.

Host plants	Site	GPS	
		North	North
1- Medicinal plants in SKP	Gebel Ahmar	28°52′83″	33°61′83″
	Wadi El-Arbaein	28°54′54″	33°55′36″
	Wadi Talaa	28°37′22″	33°52′48″
2- Mangrove plants	Port Fouad	31°14′60.00″	32°18′60.00″
	Safaga	26°43′59.99″	33°55′59.99″
	Nuwieba	28.9°73′44.4″	34.6°53′43.3″

° Degrees; ′ minutes; ″ seconds.

**Table 3:** Origin and codes of *Chaetomium* taxa.

Code	Strain	Interpretation
**1**	*Chaetomium globosum*	Soil taxon recovered from the desert in Nuwieba, South Sinai, Egypt.
**2**	*Chaetomium globosum*	Soil taxon isolated from cultivated rice straw soil, Ismailia Governorate (Suez Canal area), Egypt.
**3**	*Chaetomium globosum*	Soil taxon recovered from cultivated soil in Assiut Governorate (Upper Egypt).
**4**	*Chaetomium globosum*	Endophytic taxon recovered from *Thymus decussatus*, Gebel Ahmer, Saint Katherine, South Sinai, Egypt.
**5**	*Chaetomium globosum*	Endophytic taxon recovered from *Zygophyllum album*, Wadi El-Arbaein, Saint Katherine, South Sinai, Egypt.
**6**	*Chaetomium globosum*	Endophytic taxon recovered from *Chiliadenus montanus*, Wadi El-Arbaein, Saint Katherine, South Sinai, Egypt.
**7**	*Chaetomium iranianum*	Endophytic taxon recovered from *Origanum syriacum*, Wadi El-Arbaein, Saint Katherine, South Sinai, Egypt.
**8**	*Chaetomium globosum*	Endophytic taxon recovered from *Adiantum capillus-veneris*, Wadi Talaa, Saint Katherine, South Sinai, Egypt.
**9**	*Chaetomium globosum*	Endophytic taxon recovered from *Adiantum capillus-veneris*, Port Fouad, Port Said Governorate (Mediterranean area), Egypt.
**10**	*Chaetomium madrasense*	Endophytic taxon recovered from *Ziziphus spinosa*, Nuwieba, South Sinai, Egypt.

**Table 4:** Distribution of the microbial isolates from clinical specimens.

Organisms	No of Isolates
*Klebsiella pneumoniea*	30
*Staphylococcus aureus*	21
*Escherichia coli*	11
*Pseudomonas aeruginosa*	9
*Candida albicans*	8
*Acinetobacter baumannii*	7
*Enterococcus faecalis*	5
*Proteus mirabilis*	5
*Streptococcus pyogenes*	2
*Serratia marcescens*	2
**Total**	**100**

**Table 5:** Antibiotic resistance pattern (%) of bacterial isolates.

Antibiotics’ Classes	Antibiotic Discs	*S.* *aureus*	*E.* *faecalis*	*S.* *pyogenes*	*A.* *baumannii*	*E.* *coli*	*K.* *pneumoniea*	*Pr.* *mirabilis*	*P.* *aeruginosa*	*S.* *marcescens*
Penicillin	Amoxycillin/Clavulanic acid (30 µg)	100	60	100	57.1	90.9	100	60	100	100
	Ampicillin/Sulbactam (30 µg)	100	100	100	85.7	90.9	100	80	88.9	100
Cephalosporin	Cefepime (30 µg)	100	100	100	85.7	54.5	70	60	100	50
	Cefotaxime (10 µg)	100	100	100	71.4	81.8	90	100	100	50
	Ceftazidime (30 µg)	80.9	100	100	85.7	90.9	73.3	60	66.7	100
	Cefoxitin (30 µg)	0	100	100	100	100	90	100	88.9	50
Fluoroquinolone	Ciprofloxacin (5 µg)	71.4	80	50	85.7	81.8	80	80	88.9	50
	Levofloxacin (5 µg)	76.2	100	50	57.1	54.5	100	80	88.9	100
Aminoglycosides	Gentamicin (10 µg)	66.7	100	100	71.4	90.9	70	80	55.6	100
	Amikacin (30 µg)	76.2	100	50	71.4	54.5	73.3	40	66.7	50
Carbapenem	Imipenem (10 µg)	28.6	100	0	14.3	63.6	30	20	33.3	0
	Meropenem (10 µg)	33.3	100	0	28.6	63.6	33.3	20	11.1	0
Tetracycline	Tetracycline (30 µg)	38.0	60	50.0	57.1	63.6	80	60	100	50
	Doxycycline (5 µg)	76.2	80	50	100	72.7	60	60	100	50
	Tigecycline (15 µg)	19.0	0	0	14.3	36.4	60	20	33.3	0

**Table 6:** Antimicrobial activity of *Chaetomium* crude extracts (**1**–**10**) against clinical microbial isolates.

Crude Extracts	1	2	3	4	5	6	7	8	9	10
Microorganism	Mean of Zone Inhibition in mm (Mean ± SD)									
*Staphylococcus aureus*	0	0	19.5 ± 1.36	18.4 ± 1.4	17.2 ± 1.48	16.0 ± 1.28	0	14.8 ± 0.77	0	25.7 ± 1.23
*Enterococcus faecalis*	0	14.6 ± 0.55	0	0	0	10.4 ± 0.55	0	0	0	11.6 ± 0.55
*Streptococcus pyogenes*	0	0	21.0 ± 0.0	14.5 ± 0.7	0	15.0 ± 0.0	0	0	0	26.0 ± 1.4
*Acinetobacter baumannii*	0	0	0	0	0	15.4 ± 1.27	0	0	0	0
*Escherichia coli*	21.2 ± 2.09	19.8 ± 2.09	24.0 ± 2.19	16.0 ± 1.48	25.4 ± 2.46	15.4 ± 1.12	18.6 ± 2.46	15.4 ± 1.03	16.3 ± 0.65	10.5 ± 0.93
*Klebsiella pneumoniea*	0	0	0	0	0	0	0	0	0	0
*Proteus mirabilis*	0	0	0	0	21.6 ± 1.34	0	0	0	19.6 ± 1.34	0
*Pseudomonas aeruginosa*	26.0 ± 0.87	25.4 ± 1.24	26.3 ± 0.5	17.0 ± 1.1	21.7 ± 2.5	16.3 ± 1.1	25.2 ± 1.3	17.3 ± 0.7	21.7 ± 2.0	18.4 ± 1.5
*Serratia marcescens*	18.0 ± 0.0	16.5 ± 0.7	22.5 ± 2.1	14.5 ± 0.7	24.0 ± 0.0	0	26.0 ± 0.0	0	26.5 ± 0.7	12.0 ± 0.0
*Candida albicans*	0	0	24.5 ± 0.76	18.8 ± 1.49	25.6 ± 0.74	0	0	11.3 ± 0.7	0	15.5 ± 0.76

0, no inhibition zone.

**Table 7:** Minimum inhibitory concentration (MIC) of *Chaetomium* crude extracts (**1**–**10**).

Crude Extracts	1	2	3	4	5	6	7	8	9	10
Microorganism	MIC µg/mL (Mean ± SD)									
*Staphylococcus aureus*	-	-	29.8 ± 4.7	5.0 ± 3.5	30.5 ± 3.4	62.5 ± 0.0	-	15.0 ± 2.6	-	3.9 ± 0.0
*Enterococcus faecalis*	-	14.0 ± 3.5	-	-	-	15.6 ± 0.0	-	-	-	31.2 ± 0.0
*Streptococcus pyogenes*	-	-	31.2 ± 0.0	15.6 ± 0.0	-	15.6 ± 0.0	-	-	-	3.9 ± 0.0
*Acinetobacter baumannii*	-	-	-	-	-	31.2 ± 0.0	-	-	-	-
*Escherichia coli*	4.6 ± 1.6	8.2 ± 3.5	5.7 ± 2.0	31.2 ± 0.0	5.0 ± 1.8	29.8 ± 4.7	14.2 ± 3.2	12.8 ± 3.9	15.6 ± 0.0	14.2 ± 3.1
*Klebsiella pneumoniea*	-	-	-	-	-	-	-	-	-	-
*Proteus mirabilis*	-	-	-	-	5.5 ± 2.1	-	-	-	7.0 ± 1.7	-
*Pseudomonas aeruginosa*	4.3 ± 1.3	4.7 ± 1.7	3.9 ± 0.0	8.7 ± 2.6	5.2 ± 1.9	14.7 ± 2.6	6.0 ± 3.9	8.7 ± 2.6	15.6 ± 0.0	9.5 ± 3.4
*Serratia marcescens*	15.6 ± 0.0	15.6 ± 0.0	3.9 ± 0.0	15.6 ± 0.0	3.9 ± 0.0	-	3.9 ± 0.0	-	3.9 ± 0.0	31.2 ± 0.0
*Candida albicans*	-	-	3.9 ± 0.0	8.8 ± 2.7	4.9 ± 1.8	-	-	31.2 ± 0.0	-	14.6 ± 2.8

**Table 8:** LC-MS/MS Metabolic profiling of the crude extract of *C. globosum* soil taxon recovered from cultivated soil in Assiut Governorate (**3**).

No	Polarity Mode	MZmine ID	Ret. Time (min)	Observed *m*/*z*	Calculated *m*/*z*	Mass Error (ppm)	Adduct	Molecular Formula	MS/MS Spectrum	Deduced Compound	Ref.
1	Negative	522	1.10	173.0448	173.0450	−1.16	[M − H] ^−^	C_7_H_10_O_5_	173, 155, 111	Shikimic acid	[54]
2	Positive	201	1.11	319.1526	319.1545	−5.95	[M + H] ^+^	C_18_H_22_O_5_	319, 301, 283, 230	Zearalenone	[55]
3	Negative	663	1.15	329.0897	329.0862	10.64	[M − H] ^−^	C_14_H_18_O_9_	329, 167	Vanillic acid hexoside	[56,57]
4	Positive	885	1.34	138.0549	138.0555	−4.35	[M + H] ^+^	C_7_H_7_NO_2_	138, 94, 92	Trigonelline	[58]
5	Positive	1116	1.38	116.071	116.0712	−1.72	[M + H] ^+^	C_5_H_9_NO_2_	116, 70	L-Proline	[59]
6	Negative	1889	1.41	309.1176	309.1200	−7.76	[M − H] ^−^	C_12_H_22_O_9_	309, 89	Hex-2-ulofuranosyl-4,6- dideoxyhexopyranoside	[60]
7	Positive	1730	1.76	297.1083	297.1099	−5.39	[M + Na] ^+^	C_16_H_18_O_4_	297, 274	Prochaetoviridin A	[61]
8	Positive	1924	1.89	123.0544	123.0558	−11.38	[M + H] ^+^	C_6_H_6_N_2_O	123, 80	Nicotinamide (Niacinamide)	[62]
9	Negative	2601	3.71	351.0866	351.0869	−0.85	[M − H] ^−^	C_20_H_16_O_6_	351, 263	Altertoxin I	[63]
10	Positive	2529	4.94	314.1368	314.1381	−4.14	[M + H] ^+^	C_18_H_19_NO_4_	314, 283, 268, 251, 233, 223	Demethyl isoboldine	[64]
11	Positive	2541	5.02	286.1434	286.1443	−3.15	[M + H] ^+^	C_17_H_19_NO_3_	286, 269, 237, 209, 175, 145, 143, 107	Coclaurine	[64]
12	Positive	2609	5.34	328.156	328.1549	3.35	[M + H] ^+^	C_19_H_21_NO_4_	328, 178, 151	Scoulerine	[65]
13	Positive	2687	5.66	545.2042	545.2052	−1.83	[M + Na] ^+^	C_32_H_30_N_2_O_5_	522.60	Chaetoglobinol B	[66]
14	Positive	2747	6.08	314.1765	314.1763	0.64	[M + H] ^+^	C_19_H_24_NO_3_^+^	314, 299, 298, 269, 237, 209, 175, 121, 107	*N,N*-Dimethyl coclaurine	[64]
15	Positive	2777	6.18	500.1878	500.1840	7.60	[M + H] ^+^	C_27_H_30_ClO_6_N	500	Chaetomugilide A	[67,68]
16	Positive	2887	6.50	523.219	523.2233	−8.22	[M + H] ^+^	C_32_H_30_N_2_O_5_	523	Chaetoglobinol A	[66,69]
17	Positive	2883	6.50	300.1592	300.1600	−2.67	[M + H] ^+^	C_18_H_21_NO_3_	300, 269, 237, 209, 177, 175, 145, 107	*N*-Methyl coclaurine	[65]
18	Positive	2916	6.67	496.2383	496.2335	9.67	[M + H] ^+^	C_28_H_33_NO_7_	496	Penochalasin A	[69]
19	Positive	2921	6.69	548.2684	548.2648	6.57	[M + H] ^+^	C_32_H_37_NO_7_	548	Cytochalasin L	[70]
20	Negative	2847	6.84	523.2174	523.2177	−0.57	[M − H] ^−^	C_26_H_36_O_11_	523, 361, 346	Secoisolariciresinol *-β*-D-hexoside	[56]
21	Positive	3216	8.95	391.1363	391.1312	13.04	[M + H] ^+^	C_21_H_23_ClO_5_	391, 363, 147	Sclerotiorin	[71]
22	Positive	3337	13.47	485.2115	485.2175	12.37	[M + H] ^+^	C_27_H_32_O_8_	485	Verrucarin J	[72]

**Table 9:** LC-MS/MS Metabolic profiling of the crude extract of *C. globosum* endophytic taxon recovered from *Zygophyllum album*, Wadi El-Arbaein, Saint Katherine, South Sinai (**5**).

No	Polarity Mode	MZmine ID	Ret. Time (min)	Obseved *m*/*z*	Calculated *m*/*z*	Mass Error (ppm)	Adduct	Molecular Formula	MS/MS Spectrum	Deduced Compound	Ref.
1	Negative	56	1.08	147.0313	147.0293	13.60	[M − H] ^−^	C_5_H_8_O_5_	147, 85, 57	Citramalic acid	[98]
2	Positive	361	1.12	319.1508	319.1545	−11.59	[M + H] ^+^	C_18_H_22_O_5_	319, 301, 283, 230	Zearalenone	[55]
3	Negative	207	1.12	191.0542	191.0556	−7.33	[M − H] ^−^	C_7_H_12_O_6_	191, 173, 111, 85	Quinic acid	[99]
4	Positive	1098	1.37	433.1741	433.1762	−4.85	[M + H] ^+^	C_23_H_25_ClO_6_	433, 296	Chaetoviridin A	[100]
5	Positive	1322	1.40	138.0553	138.0555	−1.45	[M + H] ^+^	C_7_H_7_NO_2_	138, 94, 92	Trigonelline	[58]
6	Negative	2534	1.77	309.1176	309.1200	−7.76	[M − H] ^−^	C_12_H_22_O_9_	309, 179, 119, 89	Hex-2-ulofuranosyl-4,6- dideoxyhexopyranoside	[60]
7	Positive	1941	1.78	86.09572	86.0970	14.87	[M + H] ^+^	C_5_H_11_N	86, 56	Piperidine	[101]
8	Positive	2050	1.80	133.1041	133.1023	13.52	[M + H] ^+^	C_9_H_8_O	133, 115, 105, 79	Cinnamaldehyde	[102]
9	Positive	2298	1.90	402.1937	402.1919	4.48	[M + H] ^+^	C_22_H_27_NO_6_	402	Globosumin	[103]
10	Positive	2811	3.59	286.1442	286.1443	−0.35	[M + H] ^+^	C_17_H_19_NO_3_	286, 269, 237, 175, 145, 143, 107	Coclaurine	[64]
11	Positive	2959	4.41	314.1372	314.1381	−2.87	[M + H] ^+^	C_18_H_19_NO_4_	314, 283, 268, 251, 233, 223	Demethyl isoboldine	[64]
12	Positive	3163	5.30	377.149	377.1461	7.69	[M + H] ^+^	C_17_H_20_N_4_O_6_	377, 243	Riboflavin	[104]
13	Positive	3358	6.07	314.1752	314.1763	−3.50	[M + H] ^+^	C_19_H_24_NO_3_^+^	413, 299, 298, 269, 209, 175, 121, 107	*N,N*-Dimethyl coclaurine	[64]
14	Positive	3449	6.42	197.07	196.0675	12.69	[M + H] ^+^	C_7_H_8_N_4_O_3_	197, 169	1,3-Dimethyl urate	[105]
15	Positive	3505	6.54	300.1621	300.1600	6.99	[M + H] ^+^	C_18_H_21_NO_3_	300, 269, 237, 209, 177, 175, 145, 107	*N*-Methyl coclaurine	[65]
16	Positive	4060	8.10	146.0613	146.0606	4.79	[M + H] ^+^	C_9_H_7_NO	146, 117	3-Formylindole	[106]
17	Positive	4609	10.15	331.0695	331.0695	0	[M + Na] ^+^	C_17_H_12_N_2_O_4_	331, 308	Chaetominedione	[69]
18	Positive	4620	10.40	227.1166	227.1144	9.69	[M + H] ^+^	C_9_H_14_N_4_O_3_	227, 181, 156,	Carnosine	[107]
19	Positive	4628	10.51	509.2021	509.2022	−0.20	[M + H] ^+^	C_23_H_24_O_13_	509, 347	Syringetin 3-*O*-galactoside	[102]
20	Positive	5497	22.47	395.3634	395.3678	−11.13	[M + H − H_2_O] ^+^	C_29_H_48_O	395, 147	Stigmasterol	[108]

## Data Availability

The data are available within the article.
